# New-Onset Chorea Post-COVID-19 Infection: A Case Report

**DOI:** 10.7759/cureus.41327

**Published:** 2023-07-03

**Authors:** Benjamin G Grimm, Prashant A Natteru, Christopher L Groth

**Affiliations:** 1 Neurology, Roy J. and Lucille A. Carver College of Medicine, Iowa City, USA; 2 Movement Disorders, University of Iowa Hospitals and Clinics, Iowa City, USA

**Keywords:** post-acute sequelae of sars-cov-2, sars-cov-2 infection, covid-19, choreiform movement disorder, chorea

## Abstract

Although the severe acute respiratory syndrome coronavirus 2 (SARS-CoV-2) primarily involves the cardiovascular and respiratory systems, neurological manifestations, including movement disorders such as myoclonus and cerebellar ataxia, have also been reported. However, the occurrence of post-SARS-CoV-2 chorea is rare. Herein, we describe a 91-year-old female with a past medical history of hypothyroidism who developed chorea after two weeks of contracting a mild coronavirus disease (COVID-19).

## Introduction

The severe acute respiratory syndrome coronavirus 2 (SARS-CoV-2) is a novel respiratory virus that emerged in 2019 and caused a global pandemic called COVID-19. While this virus primarily affects the cardio-respiratory system, post-infectious neurological complications are not uncommon [1,2]. Though neurocognitive impairment and olfactory neuropathy are among the most common neurologic complications, meningitis, encephalitis, Guillan-Barre syndrome, strokes, and movement disorders, including myoclonus, ataxia, and action tremor, have also been reported [2,3]. However, the occurrence of post-SARS-CoV-2 chorea is rare, and to our knowledge, there have been less than 20 cases reported in the literature.

This article was previously presented as a meeting abstract at the XXVIII World Congress on Parkinson’s Disease and Related Disorders on May 15, 2023.

## Case presentation

A 91-year-old female was referred to our clinic for evaluation of abnormal involuntary movements. Six months before the evaluation, she had a bout of mild flu-like symptoms (cough, rhinorrhea, fatigue) and was diagnosed with COVID-19 after testing positive for coronavirus by reverse-transcription polymerase chain reaction (RT-PCR) nasopharyngeal swab. She did not require admission from the infection and recovered in home isolation without medical intervention. She had also received two previous COVID-19 (MODERNA) vaccines about 10 months prior to her presentation.

Two weeks after the flu-like symptoms, she developed excessive involuntary movements of the tongue, jaw, and face. Over the ensuing months, she developed excessive movements involving the arms, legs, and torso. She had no family history of a movement disorder, no personal history of anti-dopaminergic medications, tobacco, or alcohol, and her past medical history was significant only for hypothyroidism. She lived alone and was able to perform her activities of daily living before the onset of these involuntary movements. Systemic and neurological examinations were normal, except for choreiform movements in the face and bilateral upper and lower extremities, albeit her left side was more predominantly affected, as shown in Video 1.

**Video 1:** Generalized chorea albeit left hemibody predominant

**Figure 1:**
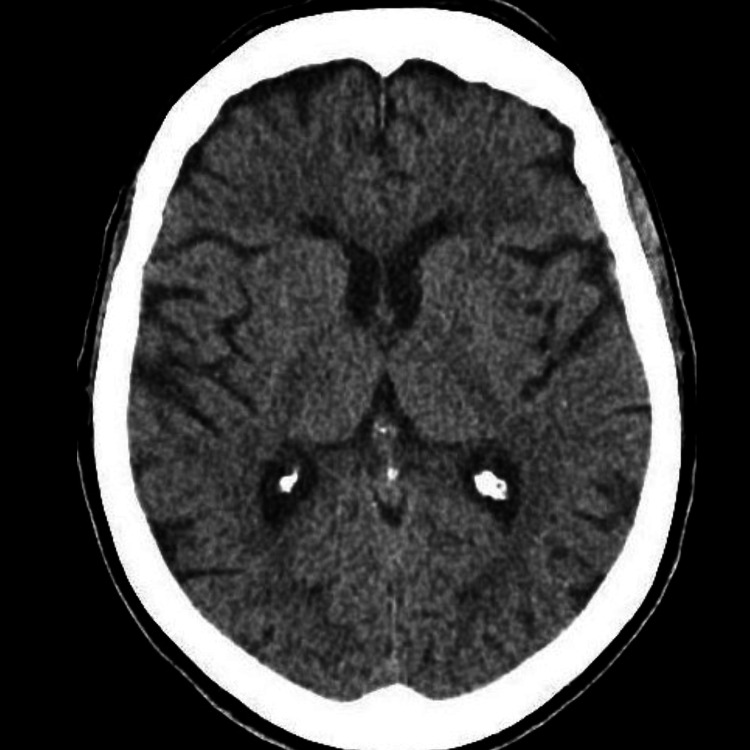
Computed tomography (CT) of the head with normal caudate and thalamus

Extensive diagnostic testing for chorea including complete blood count with peripheral smear, comprehensive metabolic panel, thyroid studies, serum paraneoplastic panels, and neuroimaging with computed tomography of the head (Figure 1) and magnetic resonance imaging of the brain was all unremarkable. Genetic testing for Huntington’s disease was not performed at the patient’s request. She was started on tetrabenazine 6.25 mg daily, six months after the onset of choreiform movements, and experienced more than 90% improvement in both facial and appendicular symptoms at one month and one-year follow-up (Video 2)

**Video 2:** Minimal chorea on follow-up

## Discussion

SARS-CoV-2 relies on the angiotensin-converting enzyme 2 (ACE-2) receptor for entry into the cells and affects the central nervous system likely through transmission via the olfactory nerve and or dissemination from the respiratory tract via the vagus nerve to the brainstem (nucleus solitarius and nucleus ambiguus in the medulla oblongata and midbrain) [2,4]. Concerning COVID-19-associated movement disorders, two mechanisms have been postulated: a) virus-induced gliosis and cellular vacuolation and b) striatal ACE-2 receptor downregulation, causing an imbalance of norepinephrine and dopamine [5].

Chorea is a hyperkinetic movement disorder characterized by involuntary, sudden, brief, and irregular movements. Sydenham’s chorea is the most common para/postinfectious entity due to autoimmunity against the basal ganglia post-streptococcal infection [1]. On the other hand, the pathogenesis of COVID-19-associated chorea is poorly understood. It is thought to be secondary to autoimmune antibodies against brain structures such as basal ganglia, on the lines of Sydenham’s chorea. Some authors have also suggested that localized hyperviscosity and focal endotheliopathy from the spike protein in the basal ganglia and thalamus contribute to neuronal dysfunction and the generation of chorea [4,6,7].

We have summarized the cases published so far with para/post-COVID-19 chorea in Table 1. The age of onset varied from eight years to 91 years, with our patient being the oldest to have developed post-COVID chorea at 91 years. In most cases, chorea developed following the onset of COVID-19 symptoms with the longest interval between the onset of chorea and COVID-19 symptoms being three months. However, there have been situations when chorea has developed along with or even preceding COVID-19 symptoms. 

**Table 1:** Cases of COVID-19-associated chorea M: Male F: Female RT-PCR: Reverse transcription-polymerase chain reaction CSF: Cerebrospinal fluid PCR: Polymerase chain reaction CRP: C-reactive protein ESR: Erythrocyte sedimentation rate SWI: Susceptibility-weighted imaging SPECT: Single-photon emission computed tomography

Author	Age/Gender	Symptom onset	Clinical features	Lab results	Imaging	Treatment	Outcome
DeVette et al. [8]	8 y.o. F	Two weeks after parents tested positive for COVID-19	Hemichorea of right arm and leg, behavioral changes, and gait instability	RT-PCR positive for COVID-19, elevated anti-streptolysin-O, anti-DNase-B	Normal	Valproate	Continued to have chorea at one-month follow-up
Ray et al. [9]	9 y.o., Not available 14 y.o., Not available	Not available	Not available	CSF studies not performed; SARS-CoV-2 IgG positive	Not available	No immunomodulation	Not available
Yuksel et al. [10]	14 y.o. F	Three days after being diagnosed with COVID-19	Bilateral shoulder shrugging, choreiform movements in all four limbs, and bilateral milkmaid's grip. History of Sydenham’s chorea three yrs ago (resolved with haloperidol)	Iron deficiency anemia	Normal	Carbamazepine	Chorea improved by the seventh day of admission
Byrnes et al. [11]	36 y.o. M	Four days prior to COVID-19 diagnosis	Homeless male with generalized chorea and mild encephalopathy	Decreased lymphocytes, SARS-CoV2 CSF PCR negative	Bilateral medial putamen and left cerebellar hyperintensities on T2-weighted imaging	IVIG, methylprednisolone	Chorea improved by day 15 with complete cessation by day 22
Hassan et al. [4]	58 y.o. M	Not known	Chorea in hands and feet	SARS-CoV-2 positivity in CSF, Leukocytosis, elevated CRP, D-dimer, and ferritin	Mild periventricular ischemic changes	Methylprednisolone, amantadine, risperidone	Improved by day 14
Ghosh et al. [12]	60 y.o. M	36 hours after onset of fever, cough, throat ache, malaise	Right-sided hemichorea-hemiballismus	Capillary glucose 540 mg/dL, ketonuria, metabolic acidosis, elevated ESR, CRP	Left striatal hyperintensity on T1-weighted imaging	Insulin for diabetic ketoacidosis	Complete resolution at six-month follow-up
Ramusino et al. [13]	62 y.o. M	Two days prior to COVID-19 diagnosis	Generalized chorea in all four limbs, head, and trunk. Mild encephalopathy	CSF PCR negative for SARS-CoV-2	Hypointense signal in the dorsolateral portion of putamen bilaterally on SWI sequence	Tetrabenazine, haloperidol	Resolution of chorea after two months from onset
Ashrafi et al. [3]	62 y.o. F	Two weeks after COVID-19 diagnosis	Choreiform movements in all limbs, predominantly on the right side	Elevated ESR, CRP	Normal	Tetrabenazine	Improvement seen; duration not available
Ashrafi et al. [3]	67 y.o. F	Three months after COVID-19 diagnosis	Random involuntary choreiform movements in her face and all four limbs, with right arm dominancy	Normal	Damaged bilateral basal ganglia	Tetrabenazine	Improvement seen; duration not available
Revert Barbera et al. [14]	69 y.o. F	Before	Mild right hemiparesis, generalized choreiform movements, seizures, and diffuse encephalopathy	Elevated D-dimer	Bilateral capsuloganglionic and thalamic infarcts. Also, with venous thrombosis of the left lateral sinus, straight sinus, and vein of Galen	Anticoagulation with enoxaparin for sinus thrombosis	Fatal from a hemorrhagic transformation of the left thalamic infarct
Our patient	91 y.o. F	14 days after the onset of flu-like symptoms	Choreiform movements in the face and all four limbs with left-side dominance	Normal	Normal	Tetrabenazine	Chorea improved 90% at one-month follow-up
Salari et al. [1]	13 y.o. M	Seven days after vaccination	Large amplitude choreiform movements on the right side	Normal	Multiple white matter lesions, one lesion enhancing with gadolinium	Intravenous methylprednisolone and tetrabenazine	Chorea improved at one-month follow-up
Salari et al. [1]	18 y.o. M	Seven days after vaccination	Choreiform movements affecting the left, shoulder, and mildly in the left leg	Normal	Few nonspecific white matter lesions	Intravenous methylprednisolone and tetrabenazine	Persistent chorea at one-month follow-up
Matar et al. [6]	88 y.o. M	16 days after vaccination	Choreiform movements in the left arm, leg, and face	Normal	Chronic small vessel ischemic change	Intravenous methylprednisolone	Resolution within 24 hours of steroid initiation
Matar et al. [6]	84 y.o. M	40 days after vaccination	Choreiform movements of left upper and lower limbs	Normal	Chronic small vessel ischemic change	Intravenous methylprednisolone	Resolution after three days of steroid initiation
Ryu et al. [7]	83 y.o. M	One day after vaccination	Choreiform movements affecting the right arm, and leg	Normal	Normal MRI, Brain SPECT with decreased perfusion in the left thalamus	Haloperidol	Resolution at two-week follow-up

Choreiform movements can be generalized or prefer one side even with a paucity of a structural lesion. With regards to COVID-19 vaccination, movement disorders’ frequency of occurrence is low (0.00002-0.0002), and tremor was the most reported side effect [2]. Salari et al. described two cases of chorea as a side effect of COVID-19 vaccination [1]. Neuroimaging can be normal but can show changes predominantly in the striatum and cerebellum [11-13]. Therapeutic options can range from tetrabenazine (most common), antipsychotics such as risperidone, haloperidol, immunomodulation with steroids, intravenous immunoglobulin, and to less common valproate and carbamazepine. Most cases have shown improvement and/or resolution with time.

## Conclusions

Even though chorea is rare, clinicians should be aware of it as a possible sequela of COVID-19 infection and in certain cases with vaccination. Our case also highlights that chorea post-COVID-19 is independent of the severity of COVID-19 infection.
